# Spatiotemporal organization of ant foraging from a complex systems perspective

**DOI:** 10.1038/s41598-024-63307-1

**Published:** 2024-06-04

**Authors:** Javier Cristín, Pol Fernández-López, Roger Lloret-Cabot, Meritxell Genovart, Viçenc Méndez, Frederic Bartumeus, Daniel Campos

**Affiliations:** 1 Istituto Sistemi Complessi, Consiglio Nazionale delle Ricerche, UOS Sapienza, 00185 Rome, Italy; 2 Dipartimento di Fisica, Universita’ Sapienza, 00185 Rome, Italy; 3 Grup de Física Estadística, Departament de Física. Facultat de Ciències), Universitat Autònoma de Barcelona, 08193 Bellaterra, Barcelona Spain; 4 Centre d’Estudis Avançats de Blanes (CEAB-CSIC), Blanes Girona, Spain; 5 CREAF, Cerdanyola del Vallès, Barcelona, Spain; 6 ICREA, Institut Català de Recerca i Estudis Avançats, Barcelona, Spain

**Keywords:** Ecological modelling, Biological physics, Statistical physics

## Abstract

We use complex systems science to explore the emergent behavioral patterns that typify eusocial species, using collective ant foraging as a paradigmatic example. Our particular aim is to provide a methodology to quantify how the collective orchestration of foraging provides functional advantages to ant colonies. For this, we combine (i) a purpose-built experimental arena replicating ant foraging across realistic spatial and temporal scales, and (ii) a set of analytical tools, grounded in information theory and spin-glass approaches, to explore the resulting data. This combined approach yields *computational replicas* of the colonies; these are high-dimensional models that store the experimental foraging patterns through a training process, and are then able to generate statistically similar patterns, in an analogous way to machine learning tools. These in silico models are then used to explore the colony performance under different resource availability scenarios. Our findings highlight how replicas of the colonies trained under constant and predictable experimental food conditions exhibit heightened foraging efficiencies, manifested in reduced times for food discovery and gathering, and accelerated transmission of information under similar conditions. However, these same replicas demonstrate a lack of resilience when faced with new foraging conditions. Conversely, replicas of colonies trained under fluctuating and uncertain food conditions reveal lower efficiencies at specific environments but increased resilience to shifts in food location.

## Introduction

Ant colonies, along with other eusocial species, serve as a paradigmatic example of complex systems and collective search dynamics^1–4^. Ant societies exhibit emergent behaviors and local mechanisms that interconnect various scales of organization, aligning with foundational concepts of complexity theory ^5,6^. The levels of collective organization and order in ants in the absence of centralized leadership or a hierarchical governance structure^4,7,8^ is remarkable. Beyond the confines of traditional neural systems, ant colonies establish and sustain fluid agent-to-agent communication networks over the long term. Such *liquid brains* are expected to have evolved in order to optimize the fitness of the group ^9^.

In a social context, foraging efficiency is presumably governed by both the number of individuals involved in the process, and the extent of interactions among them. Information is stored, processed, transported, and transmitted through the colony by individual agents in motion, capable of interacting with one another. Therefore, individual memory and movement modulate interactions, and viceversa, interactions may shift movement behaviours and update memory. To bring a complex systems approach to topic of collective foraging, it is essential to identify appropriate measures of complexity and adapt them to this particular context, a matter currently under active debate^10,11^. At present, biologists studying the complexity of insect colonies employ a diverse array of measures and proxies, which are context-dependent. However, many of these measures predominantly focus on capturing aspects of social organization^12,13^ and the intensity and diversity of social interactions within the group^14^. In the case of ants, long-term interest has been directed toward comprehending task allocation within colonies^4,15^ and exploring its potential functional advantages^16,17^. Importantly, other research ^18–20^ has explored the transition from individual to collective foraging, representing an example of emergent behavior in ant colonies.

Physicists and theoretical researchers from the field of complex systems have also made their contributions, particularly in understanding the formation of pheromone trails^21,22^ and the rules governing collective movement along these trails^23,24^. However, despite efforts to facilitate the exchange of ideas between communities from complex systems theory and social ecology (see, e.g.,^25–28^), substantial cultural barriers persist.

In the current article, we aim to strengthen the connections between biology and statistical physics, with a specific focus on the occupancy patterns observed during ant foraging processes, and understanding their significance in terms of foraging efficiency at the colony level. For this purpose, we specifically characterize and elucidate the relationships between two key elements: (i) the temporal regulation of energy investment in foraging by ant colonies, measured through the number of individuals exiting the nest, and (ii) the spatial distribution of this investment, quantified through the spatial correlations in occupancy patterns. These correlations are, to a great extent, a result of the communication network within the colony.

To address the questions at hand, our methodology integrates (i) experiments conducted in the laboratory at ecologically realistic scales, with (ii) the utilization of spin-glass models as computational tools. Spin-glass models are particularly well-suited for capturing spatial (pairwise) correlations inherent in highly-dimensional datasets^29,30^. When provided with experimental data from ant behavior (through a training process equivalent to that used for neural networks and similar tools for machine learning), spin-glass models are able to implement the properties of the patterns observed, and so they can be understood as *computational replicas* of the ant colonies, that is, models able to generate new datasets (patterns) mimicking those properties^29^. These replicas are valuable tools for conducting in silico experiments in which the efficiency of synthetic foraging patterns can be explored, enabling us to computationally predict the performance of different ant colony organization under distinct resource conditions. This approach serves then as an indirect method to offer quantitative evidence regarding the functional and adaptive advantages of foraging behaviour at the colony level.

The present article is organized as follows. In “Materials and methods”, we present the setup and methods used for collecting our experimental data, and the theoretical tools/approaches employed for its subsequent analysis. In “Experimental patterns of ant foraging”, we quantify the spatial correlations in the foraging patterns of ants as a first approximation to understand how ants interact during foraging. For this, we first use a more conventional approach based on assessing the foraging activity, that is, the number of ants coming out of the nest and the encounters between them, and then we refine this analysis by using the mutual information between spatial regions of the arena. The data and conclusions gained from this analysis is finally used in “Spin-glasses can predict ant colony foraging” to build our computational replicas of the ant colonies (from spin-glass models) and create computer simulations of ant foraging under different conditions. Finally, “Discussion” wraps up the conclusions drawn from our study.

## Materials and methods

### Experimental methods

**Figure 1:**
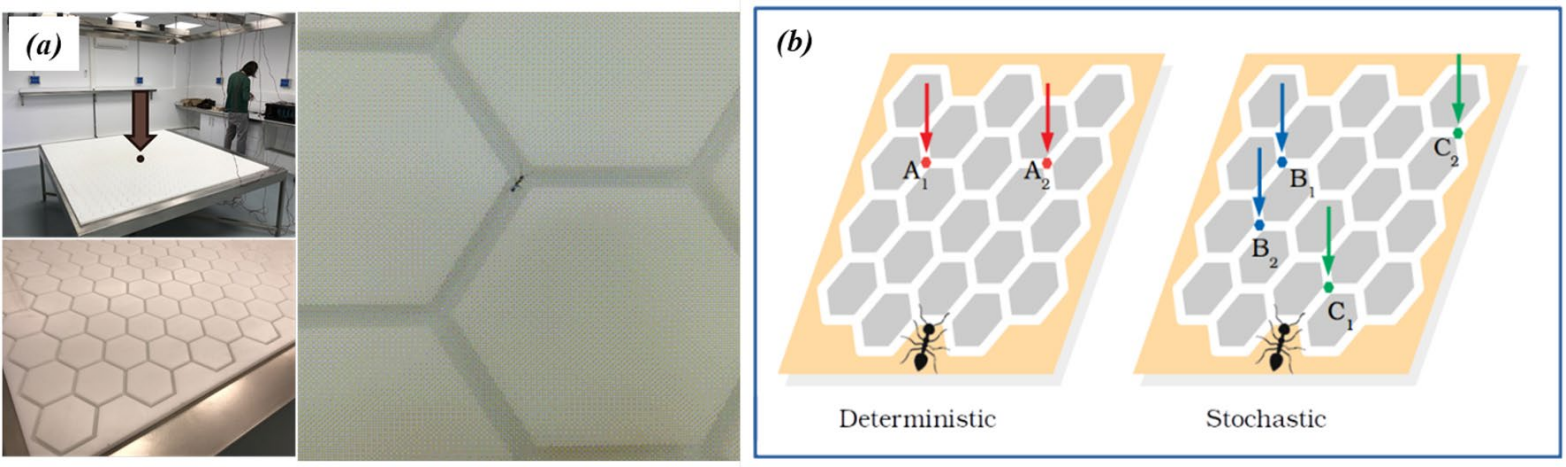
(**a**) Experimental setup (left: general view, right: detailed view of the nodes and hexagons) used to track foraging trajectories in an hexagonal lattice of 2 m^2^. The ants nest is connected to the structure from below through a plastic tube, the access point to the arena is marked with a grey arrow/cicrle (note that only half of the total lattice seen is explored by each colony, so at practice two experiments can be carried out simultaneously with two different colonies, separated by plastic barriers in the lattice). (**b**) Schematic representation of the food location. In the left panel, the scenario (deterministic) in which both worm pieces are placed in the same patches in each daily experiment ($A_{1}$ and $A_{2}$). In the right panel , the scenario (stochastic) in which both worm pieces are placed in patches selected at random for each experiment. Blue and green dots corresponds to the resource location for a given daily trial *i* and the next one $i+1$, respectively.

We obtained two gypsy ant colonies (*Aphaenogaster senilis*) from the field, with about 750 individuals each. At the lab, we held the colonies isolated in independent plastic structures, with an opaquely covered region in it, as a nest. We connected the nests to a large arena, and we let each colony to explore half of it, in order to find and collect food located at targeted or random locations. The arena follows an hexagon tessellated pattern (see Fig. 1). This system allowed us to (i) reduce the movement degree’s of freedom and analyze movement as an Eulerian process (node focus), and (ii) constrain the minimum spatial scale (the distance between neighbouring nodes) of the foraging process. Our observations covered a wide range of temporal scales (from 0.5 seconds to 3 h) enabling us to cover behavioural processes from the individual to the population level.

The experimental arena consisted on a squared Teflon structure of $2 \times 2$ m^2^, with 2 cm of height. Channels of 5 cm length and 7 mm width (the wingspan of ants antennae) were mined in the Teflon structure to conform 572 hexagons (Fig. 1a). Each hexagon vertex defines a decision-making node where left, right or backwards directions can be chosen. There are 1192 of such Y-maze crossings, 1240 including the peripheral ones with only forward-backward binary directional options. To perform experimental replicates with the two ant colonies (colony *A* and *B* respectively), the arena was divided into two rectangular independent sections (of $2 \times 1$ square meters each), the actual foraging area for each of the colonies in our experiments. This foraging area contained an hexagonal lattice of 286 hexagons and 596 y-maze crossings (if we do not consider the nodes at the perimeter border). Colony nests were located below the arena and connected through a tube (one for each colony) to an orifice located at the centre of the arena that served as point of entrance and departure. To avoid ants escaping from the channels we covered the whole structure with a well-stretched and framed mosquito net, so that ant movement was restricted only to the channels. The movement of all individuals was recorded with a synchronized network of 12 cameras working at 2 Hz and 12 MP of resolution, placed 1 metre above the arena. Each camera was surrounded by an annular-led light providing zenital and homogeneous illumination. The arena was also surrounded by dark-blue colored sheets to absorb light scattering and reflections. Room temperature was set at 25 ^∘^C.

The foraging experiments were carried out using three different food scenarios/conditions (see Fig. 1b). In the so-called *deterministic* scenario or condition the food nodes were always the same; in particular, we introduced 12 pieces of food (small pieces of the worm *Tenebrio sp.*) at 12 nodes forming the vertex of two targeted hexagons, placed at a mid-range distance from the nest. For the *stochastic* condition, instead, the two patch-hexagons where the food was placed were chosen randomly among all those present in the arena. Finally, we carried out experiments under a *no food* condition as a control case against which to compare the two previous ones.

Once food was put into place (for deterministic and stochastic conditions) we connected the nests to the arena and during 3 hours (i.e. 10, 800 s), we captured a picture of the arena at a rate of 2 Hz. During this time span, the ants moved freely within the arena searching for food and exploiting the food resource by means of the group recruitment strategy^31,32^, where scouts that found the food came back to the nest to recruit 3–5 individuals in order to bring other food pieces to the nest.

We performed this experiment two days a week for five weeks, running two experiments per day (morning and afternoon, for the two colonies *A* and *B* simultaneously). In the deterministic conditions, we never cleaned the arena, so that ant cues would remain in the system on the next trial. In the stochastic scenario we cleaned with alcohol the arena and let it dry before starting the next trial. For the first *two and a half* weeks of experiments, the resources were placed as presented in deterministic conditions, which, according to our observations and previous experience with similar foraging experiments^29,32^ is a large enough time for the colony to get adapted. Then, during the next *two and a half* weeks food was located in the maze following stochastic conditions. We observed that colony *A* seemed to explore more actively the discrete arena than the colony *B*. In spite of this fact, both colonies obtained the worm pieces during all the experimental trials.

In total, we performed 20 experiments (i.e. 10 experiments for each condition). However, we had to discard 4 experiments (three stochastic and one deterministic) for various reasons like malfunctioning of the video recording or mistakes during experimental procedures (e.g. lack of food in one of the patches in one of the cases). In addition to the foraging experiments, we also let the ants explore the arena without food on it, i.e. 12 runs of no-food condition.

Images of each camera were stitched into a single video file, encompassing the whole arena. This process was performed in two steps: (i) calculating homography for each image (i.e. placing the coordinates of the pixels in the same plane) and (ii) finding and cutting the overlapping regions of the individual images. From the stitched video file, we detected ant positions using a background subtraction approach, by (i) converting the image to grey-scale, (ii) converting the image to black and white, according to a pixel value threshold, (iii) performing some computer vision operations (i.e. eliminating noise and joining blobs) and (iv) subtracting the pixel values of each image to a background frame (i.e. an image of the arena with the same illuminating conditions, and without ants). The centroid of the detected ants (contours) were calculated and written in a CSV file with the pixel coordinates and the corresponding frame. These coordinates were converted to millimeters (applying a factor mm/pixel), and frame number converted to time (according to the framerate of 2 Hz). Ant activity was then computed as the number of individuals present in the lattice, and ant interactions (which are computed through ant-ant encounters taking place within the lattice/arena) were approximated using different criteria (i.e. distance between two blobs less than a body length, or loss and gain in number of blobs in regions other than the nest) to check the robustness and validity of our results. Both measures, activity and interactions, were computed at each frame.

### Information theory and complex systems

#### Mutual information

The occupancy of each node of the arena can be defined through a binary variable $I_i(t)$ (where *i* represents the index of the node) which equals 1 in case the node is occupied at time *t*, or 0 otherwise. The overall occupancy is then given by the multidimensional set $\varvec{I}(t)={I_1 (t),I_2 (t), \ldots , I_N (t)}$, where $N=620$ is the number of nodes, so $\varvec{I}(t)$ can take $2^{620} \approx 10^{186}$ different states/configurations.

To capture how much of the information contained within $\varvec{I}(t)$ is really due to collective organization one can explore the correlations that appear between the occupancies at the different nodes in the arena. Alternatively, information theory prescribes that the mutual information between variables $I_i$ and $I_j$ provides the amount of information present in the variables that cannot be captured thorugh their analysis by sepparate. We note that this has the advantage of providing a quantity that can be further compared and studied with other information-theoretic measures in potentially futures analysis of the dataset. For this reason, in the present work we will adopt this perspective and will characterize collective foraging patterns through the MI defined as

1 $$\begin{aligned} \text {MI} (I_i,I_j)= \sum P_{ij}(I_i,I_j) \log {\frac{P_{ij}(I_i,I_j)}{P_i(I_i) P_j(I_j)}}, \end{aligned}$$

where $P_i$, $P_j$ correspond to the probability distribution of the variables $I_i$ and $I_j$, respectively, and $P_{ij}$ is their joint probability distribution. Note that we measure so the information contained within spatial regions, taking a space-use perspective (rather than an individual-based approach) as this is more informative of the patterns emerging at the scale of the spatial domains considered. The convenience of site-based over individual-based approaches in similar modeling contexts has actually be discussed in recent works (see^33^ and references there in).

For computing overall correlations between all nodes of the lattice/arena, however, a unique direct generalization of mutual information to the case of multiple variables does not exist. One option is then to carry out an average over the mutual information of all the pairs of variables in the system:

2 $$\begin{aligned} \langle \text {MI} \rangle \equiv \frac{2}{N (N-1)} \sum _{i \ne j} \text {MI} (I_i,I_j) \end{aligned}$$

Importantly, this measure does not take into account higher-order (triplet, quadruplet, ...) correlations in the system. To include such additional information alternative measures have been explored in the literature (see^34,35^). However, for the focus of the present work we will keep our level of analysis up to the case of pairwise interactions, that has been observed before to be enough to capture foraging spatiotemporal patterns in similar experimental datasets^29^.

#### Inverse spin-glasses

A spin-glass model consists of a set of discrete units (spins) which are often restricted to behave as binary variables with explicit pairwise interactions among them. Gradually, these models have become a paradigm of complex systems, also in biology^30,36^, and have been increasingly used as a way to understand underlying interactions in collective systems of very different nature, as the spiking of groups of neurons^37^, flocking dynamics of birds^38^ or human social networks^39^. Recently, some of us have illustrated how they can be also used to characterize space use of biological species^29^.

In the spin-glass framework, we define the probability to find the system in a particular occupancy state $\varvec{I}(t)={I_1 (t),I_2 (t), \ldots , I_N (t)}$ through a canonical probability distribution

3 $$\begin{aligned} P^{(1:N)}(\varvec{I}) = \frac{1}{Z} \text {exp} \left[ \beta \left( \sum _{i=1}^{N} h_i \sigma _i + \sum _{i \ne j} J_{ij} \sigma _{i} \sigma _{j} \right) \right] , \end{aligned}$$

where *Z* is a normalization factor, $\beta$ is a free parameter (in our case we take $\beta =1$ unless specified otherwise), the sum $\sum _{i \ne j}$ extends over all pairs of spins (or variables) in the system, and we have redefined the occupancies through $\sigma _i \equiv 2I_i-1$, to take values $\sigma _i = \pm 1$, as is assumed by default in most spin-glass frameworks. Finally, $h_i$ and $J_{ij}$ are parameters that measure, respectively, the weight of each variable $\sigma _i$ and each pair $(\sigma _{i}, \sigma _{j})$ on the probability distribution. This means that a high value of $h_i$ correlates with a higher occupancy of node *i*, while a high value of $J_{ij}$ correlates with a high correlation between occupancies of nodes *i* and *j* (see Section S2 in the Supplementary Material, and Ref.^29^ for a quantitative account on this).

One can use Eq. (3) to recover the dynamics and the correlations of the spatial distributions of a dataset through the so-called *inverse Ising problem*^29,40^. This is, given the set $\varvec{I}(t)$ obtained experimentally, one can use standard techniques to infer the values of the parameters $h_i$ and $J_{ij}$ that maximize the log-likelihood function $\mathcal {L}=-\log {P^{(1:N)} (\varvec{I})}$ (a detailed compilation of the numerical methods available is provided in^29,40^ and the references there in). The resulting set of parameters provides a computational model, or *replica*, of the colony that is able to reproduce in detail its spatial dynamics. This can be confirmed *a posteriori* by generating synthetic datasets through Monte Carlo simulations with Eq. (3), and checking that the corresponding artificial occupancy patterns closely resemble the experimental ones (see Section S3 in the Supplementary Material for a detailed study applied to our particular case).

### Null model

Finally, note that as $J_{ij}$ contains the information about correlations between the occupancy of the different spins (nodes), if we force $J_{ij}=0$ in Eq. (3) (so we use inference only to determine the $h_i$ parameters) then we obtain replicas of the colony which do not include spatial correlations. These particular replicas would produce spatiotemporal patterns that reproduce the experimental occupancies $\langle I_i \rangle$ at each node, but not their collective dynamics. Comparing the case $J_{ij}=0$ to the general case, one can then explore what is the effect that such collective dynamics really play in the organization of foraging.

## Experimental patterns of ant foraging

###  The three foraging phases in ant activity

The experimental data uncovered a non-stationary foraging dynamics which can be characterized (as a first approximation) through the existence of two significant Transition Points (TP) that are used as time boundaries associated with the beginning/end of significant phases in the foraging process. The first Transition Point (TP1) centers around the time when ants discover the first food item, while the second Transition Point (TP2) corresponds to the time when ants have collected the last food item in the arena, signifying the depletion of all available food resources in that space.

**Figure 2:**
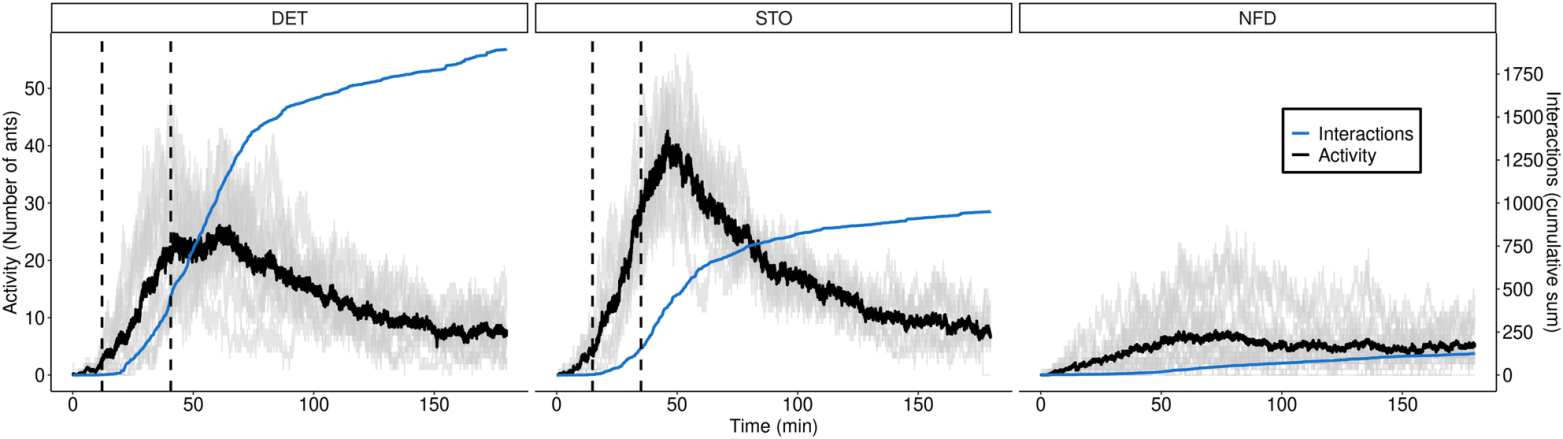
Averaged experimental measures of ant activity (number of ants in the arena) and cumulative interactions for deterministic (DET), stochastic (STO) and absence of food (NFD) scenarios. Ant activity measured at each frame is shown along the three stages described in the main text: exploration, exploitation and relaxation. The dashed lines show average values for two key Transition Points (TP1: first food item detected, TP2: last food item collected) separating the foraging dynamics in three phases: exploration (from initial moment to TP1), exploitation (from TP1 to TP2), and relaxation (from TP2 until the end of experiment). The black curve represents the averaged occupancy across all experiments in the corresponding experimental condition. The blue curves show the cumulative number of interactions (i.e. ant-ant encounters), also measured at each frame. Grey curves depict ant activity for each individual experiment.

The period from the beginning of the experiment until TP1 is denoted as the *exploration* phase, characterized by a relatively small group of scout ants exploring the arena. When a scouting ant discovers a resource (TP1), a consistent and substantial change in population numbers is triggered (see Fig. 2). Ant numbers and interactions increased from 5 to 10 fold over a relatively short period of time of 35 minutes on average. This phenomenon occurs independently of the experimental food condition, whether deterministic or stochastic. As known in the literature^41^, the ant that finds food returns to the nest to recruit other colony workers, leading to a notable increase in ant numbers and interactions, a process commonly referred to as *group recruitment*.

The increased activity during group recruitment and food gathering leads to the formation of ant trails connecting the food sources with the nest, and vice versa. This heightened activity continues until the collection of the last food item (TP2), signifying the conclusion of the *exploitation* phase. Importantly, during this phase, not all ant trajectories are confined to the nest-to-food trails and back; some ants exhibit marginal exploratory behavior, further contributing to the dynamic foraging process.

After TP2, the system gradually returns to a relaxed state, leading to a decrease in ant activity in the arena down to 5 to 10 ants, in average. This period, labeled as the *relaxation* phase, is characterized by waves of nest-departure activity bursts combined with an overall damping trend of ant numbers in the arena. Activity bursts during the relaxation phase are a typical feature of the foraging dynamics of *A. senilis* and other ant species^42,43^, representing a large-scale manifestation of the heterogeneity in the response rate of individuals to local interactions^4,44^ .

While the three identified phases (exploration, exploitation, relaxation) remained consistent and qualitatively similar in both deterministic and stochastic scenarios, some differences emerged. Notably, higher ant activity, reflected in the number of individuals, was observed in stochastic experiments across all three phases. This suggests that ants may compensate for the inherent lack of information in stochastic environments by increasing their occupancy on the lattice, potentially placing more emphasis on exploration.

Conversely, ants exhibited more interactions under deterministic food conditions than in stochastic conditions. In the former case, most individuals concentrated in a smaller region, a result of bidirectional ant flows linking resource patches with the nest. On average, the total number of interactions accumulated in deterministic foraging conditions was twice that of stochastic foraging conditions. This result can be complemented by exploring the average interactions and the occupancy “per capita”, measured as the proportion of the number of nodes occupied divided by the number of ants in the arena at each frame, and then averaging for each phase and condition; this analysis is provided in the Supplementary Material. According to it, ants tend to interact more in DET experiments during the exploitation and relaxation phases, but not in the exploration phase. However, no differences seemed to occur in the occupancy “per capita” between the exploitation and relaxation phases.

### Spatial patterns of mutual information

**Figure 3:**
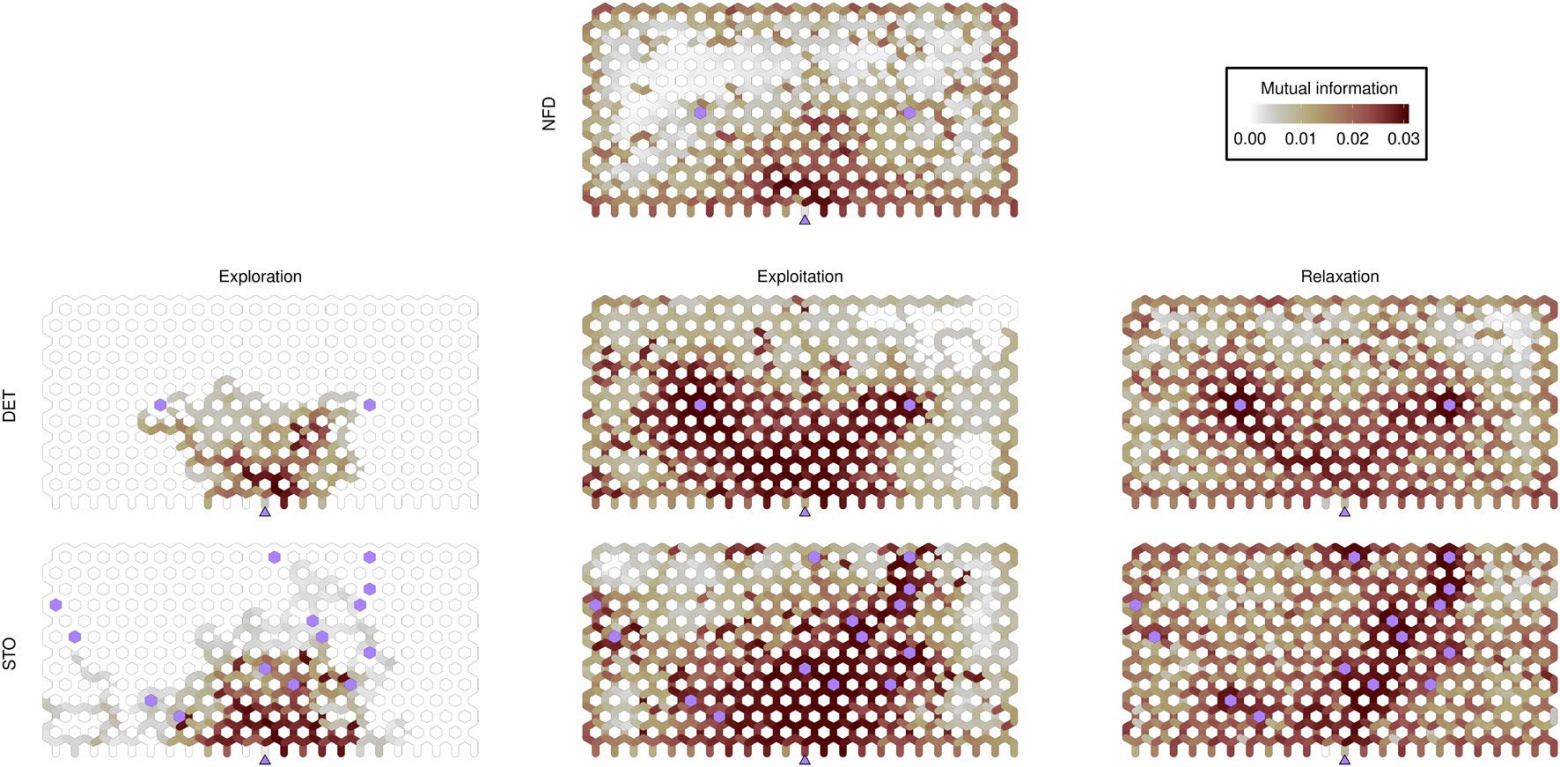
Heatmaps of averaged mutual information patterns (nearest neighbors mutual information) for deterministic (DET), stochastic (STO) and absence of food (NFD) scenarios along the three phases of foraging: exploration, exploitation and relaxation. Note that the NFD condition is considered exploration behaviour. Purple filled hexagons show the location of resource patches. The purple triangle (in the bottom center of the lattice) corresponds to the entry point from the nest to the arena. Colormap (top right): Mutual information from high (dark red) to low (white) values.

We quantified the spatial occupancy patterns in each of the three foraging phases, and for the three food conditions explored (Fig. 3), based on the computation of the local (nearest neighbors) average mutual information. We found: (i) an initial occupancy centered around the nest during the exploration phase, (ii) transition to paths connecting the nest to food sources during the exploitation phase, with some local exploration around food patches, and (iii) a gradual decay in occupancy around patches, leading to increasingly homogeneous spatial patterns until the end of the experiment (relaxation phase).

The mutual information, accordingly, reflect these behavioral patterns, and specifically it shows the emergence of the persistent paths during the exploitation phase, which the colony consistently utilizes for food collection. Notably, the presence of pheromone trails is expected to be minimal, as *A. senilis* relies on pheromone trails primarily for food sources above a critical size or mass^31^—a threshold much higher than the food targets in our experiments. Therefore, the persistent paths observed likely result from a combination of antenna contacts, visual cues, and nest mate odor gradients, all contributing to the established communication network of the colony.

Additionally, the mutual information in Fig. 3 reveals, as usual in bounded domains, the existence of thigmotactic effects (this is, a trend of the individuals to follow the boundaries of the domain) that become more noticeable as the region explored by the colony increases (and especially for the case without food, NFD, due to the absence of trails in that case).

Remarkably, the spatial patterns observed also showed differences between deterministic and stochastic conditions. In stochastic scenarios there were more extensive patterns compared to the deterministic scenarios. In the latter, occupancy and the corresponding correlations are mostly concentrated in a limited area, specifically in nodes that form part of nest-to-food trails. This observation reflects that exploratory behaviour is more pronounced and enduring in the stochastic food condition, meaning also less structured and dense nest-to-food trail formation.

### Temporal patterns of mutual information

**Figure 4:**
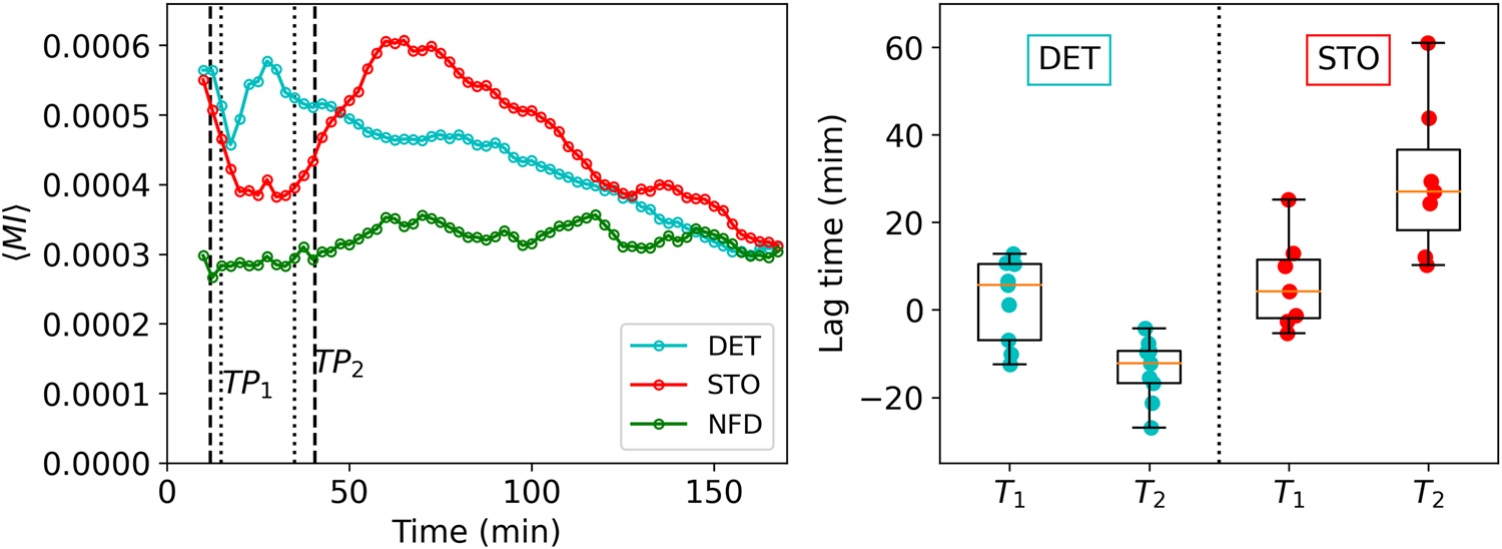
(Left) Evolution of the average mutual information between the occupancies of the different pairs of nodes in the lattice during the experiment for the deterministic, stochastic and control (no food) conditions. Symbols shown correspond to values obtained every 2.5 min for a moving time window of 20 min. the vertical lines correspond to the average of the two transition points $TP_1$ and $TP_2$ obtained for the deterministic (dashed lines) and stochastic (dotted lines) conditions. (Right) Boxplot of the response times $T_1$ and $T_2$, as defined in the main text, obtained for the deterministic and stochastic food conditions (see legend). Open symbols correspond to the whole set of values obtained in each single experiment.

We further investigated the temporal evolution of the averaged mutual information $\langle \text {MI} \rangle$ over time (Fig. 4, left), utilizing a moving time window of 15 minutes to mitigate noise. Both deterministic and stochastic conditions demonstrate fluctuations in the temporal evolution of the mutual information, aligning with the different phases identified. During the exploration phase, there are fewer ants in the arena, and their trajectories are largely independent, resulting in a decrease in $\langle \text {MI} \rangle$. Following the exploitation phase, ants formed nest-to-food trails, leading to an increase in $\langle \text {MI} \rangle$ due to the concentration of occupied nodes in a relatively small region. As food was collected, and the trails were less used, $\langle \text {MI} \rangle$ decreased again as ants dispersed through the arena.

This interpretation was reinforced by the behavior observed in the control case (no food), where these distinct phases are absent. With no food, mutual information remained approximately constant, at values similar to those obtained for the deterministic and stochastic food conditioned experiments at the end of the relaxation phase (Fig. 4, left).

All in all, the mutual information variation reflects the exploration-exploitation dynamics during the foraging process. Periods with an increase in $\langle \text {MI} \rangle$ should be associated to the emergence of more rigid/systematic behavior (so to a dominance of exploitation), while a decreasing $\langle \text {MI} \rangle$ indicates the tendency of ants to adopt more dispersed (off-trail) trajectories (promoting exploration). Then, ideally the transition point TP1 (first food item discovered) should lead to a period of increasing $\langle \text {MI} \rangle$, whereas TP2 (last food item collected) should promote a gradual decrease in $\langle \text {MI} \rangle$. At practice, however, TPs and Mutual Information trends are not fully synchronized (Fig. 4, left panel; compare dashed vertical lines with $\langle \text {MI} \rangle$ evolution). To gauge the time lag response of Mutual Information, we introduced lag times $T_1$ and $T_2$: we define $T_1$ as the time at which $\langle \text {MI} \rangle$ begins to increase minus the time at which the first food item is discovered (TP1). $T_2$ is the time at which $\langle \text {MI} \rangle$ starts to decrease minus the time at which food collection concludes (TP2).

The boxplot in Fig. 4 (right) illustrates the values of the lag times obtained from each individual experiment in deterministic and stochastic conditions. Negative values indicate temporal mutual responses anticipated to the TPs, while positive values represent temporal responses delayed from the TPs. The first-detection event TP1 triggered a relatively fast response (increasing $\langle \text {MI} \rangle$) under both food conditions, with an average $T_1$ of around 2 and 3 min for the deterministic and stochastic cases, respectively. Experiment-to-experiment variability is notable, with $T_1$ occasionally taking slightly positive or negative values, particularly in the deterministic case. Some negative values of the time lags were observed, suggesting a learning process where colonies are strongly preconditioned, so automatically concentrating foraging efforts (including trail formation) on the successful paths identified in previous trials/experiments.

In contrast, the values of $T_2$ revealed a substantial difference between deterministic and stochastic food conditions. In deterministic experiments, the tendency of $\langle \text {MI} \rangle$ to decrease starts before food collection is completed, resulting in negative $T_2$ values. The deterministic colonies, adapted to a constant foraging scenario, presumably predict in advance that food will be depleted, prompting an automatic reactivation of exploration to seek additional food. Conversely, colonies experiencing stochastic environments consistently exhibit positive $T_2$ values, indicating a prolonged time before abandoning the exploitation phase. Our intuition is that this delay has probably different origins. First, the absence of paths and clear expectations about the food locations for the stochastic case makes that, even when the first patch/resource is found, paths are not easily created, and exploration is still largely sustained as it is required to detect additional sources (while in the deterministic case finding the first resource facilitates the formation of a systematic path to the second one). Second, as a result of this dynamics it is likely that communication between individuals in the stochastic case is slower and less fluid due to fluctuating food conditions, especially in those trials where food locations are distant from the nest. Also, note that the peak reached by the $\langle \text {MI} \rangle$ after TP2 is larger for the stochastic condition. Again, this is due to the larger uncertainty associated to this case, which leads in general the colony to explore further regions of the lattice, and so the average mutual information contains contributions from a larger number of nodes.

In summary, these findings indicate that the colony adapted to a deterministic food condition exhibits faster responses in relation to information obtained in the two TPs (at the expense of sacrificing exploration by reducing the total area explored, as evidenced by smaller $\langle \text {MI} \rangle$ values). Conversely, the colony adapted to stochastic food conditions displayed larger and always positive $\langle \text {MI} \rangle$ response times as a result of more dispersed and variate exploration pattern, as well as delayed reactions to the TPs as a consequence of a smaller reliance on collective trails for exploitation.

We note that our conclusions about the performance under deterministic vs stochastic conditions are essentially qualitative up to now. So, next step then would be to identify tools and methods to quantify how enhanced efficiency emerging under the deterministic condition comes at the expense of lower flexibility to external changes; this is undertaken in the next Section.

## Spin-glasses can predict ant colony foraging

The observation that $\langle \text {MI} \rangle$ appears to capture substantial information about the spatiotemporal foraging patterns suggests that pairwise interactions might be sufficient to replicate, at least to a preliminary extent, these experimental patterns (disregarding higher-order correlations between the nodes occupancies). Specifically, we find that pair correlations account for between 40$\%$ and 96$\%$ of the total correlations in the system, depending on the food conditions and/or the phase considered (see Section S3 in the Supplementary Material for details about how we quantify these effects). This prevalence of pairwise correlations echoes similar findings in other studies on animal or social networks^37,39^, and its implications and significance are currently subjects of debate^45,46^.

These results prompt the question: *Can we propose a basic model based on pairwise correlations (for simplicity) to replicate the foraging dynamics of ant colonies for predictive purposes?* Drawing inspiration from the literature in complex systems, spin-glass models^30,36^ emerge as promising candidates for this. Similar to neural networks^40,47^, these models can be trained to emulate the properties of experimental datasets, effectively serving as computational *replicas* of the colonies. These replicas can then be leveraged to generate synthetic foraging trajectories and patterns that statistically reproduce the mean occupancy and pairwise correlations observed experimentally^29^.

To comprehend the intricate dynamics of foraging patterns within the context of spin-glasses, we conducted a preliminary analysis, similar to that carried out for a simpler experimental setup in a previous work^29^; the details are provided in the Supplementary Material file. This analysis involved a comparative examination of three distinct sets of trajectories:

- Experimental trajectories: reflecting real-world foraging behavior.
- Synthetic trajectories from general spin-glasses: artificial trajectories generated based on the characteristics of general spin-glasses.
- Synthetic trajectories with suppressed spatial correlations (null model): artificial trajectories created under the condition where spatial correlations and collective effects are intentionally ignored (indicated by $J=0$).

Our focus during this analysis was on studying both occupancy patterns and mutual information patterns. Trajectories derived from general spin-glasses remarkably capture the statistical patterns of foraging observed in experimental trajectories. This accuracy holds true across both temporal evolution and spatial distribution within the arena. In stark contrast, synthetic trajectories under the condition of suppressed correlations ($J=0$) fail to replicate the observed foraging patterns. This discrepancy emphasizes the pivotal role played by correlations in shaping these behavioral patterns. These findings then underscore the significance of spatial correlations and collective effects in understanding and replicating observed foraging behaviors.

### Impact and correction of the overall occupancy

The introduction of spatial correlations in the spin-glass model yields a dynamics where the occupation of specific nodes in the arena becomes linked to the occupation of other regions, particularly if the correlations are predominantly positive (as observed in our datasets). In this context, the general spin-glass case is then expected to exhibit a somewhat higher ant occupancy compared to the scenario where spatial correlations are completely absent ($J=0$). In other words, positive correlations imply a cooperative behavior among ants, where the presence of ants in one node positively influences or triggers the occupation of nearby regions. This interconnectedness fosters a more coordinated and collective response during foraging. Hence, these positive spatial correlations are likely to result in a higher overall ant occupancy compared to the case where correlations are suppressed ($J=0$), and we need to control for this aside effect when using spin-glasses as a *replica* of the experimental colonies.

To scrutinize potential biases, we have examined the overall occupancy patterns generated by different *replicas*. Notably, the spin-glass framework allows easy tuning to either augment or diminish occupancy (mean number of occupied nodes) by adjusting the parameter $\beta$ in Eq. (3). It is noteworthy that this adjustment does not significantly impact foraging patterns, provided a relatively narrow range of $\beta$ values is used.

**Figure 5:**
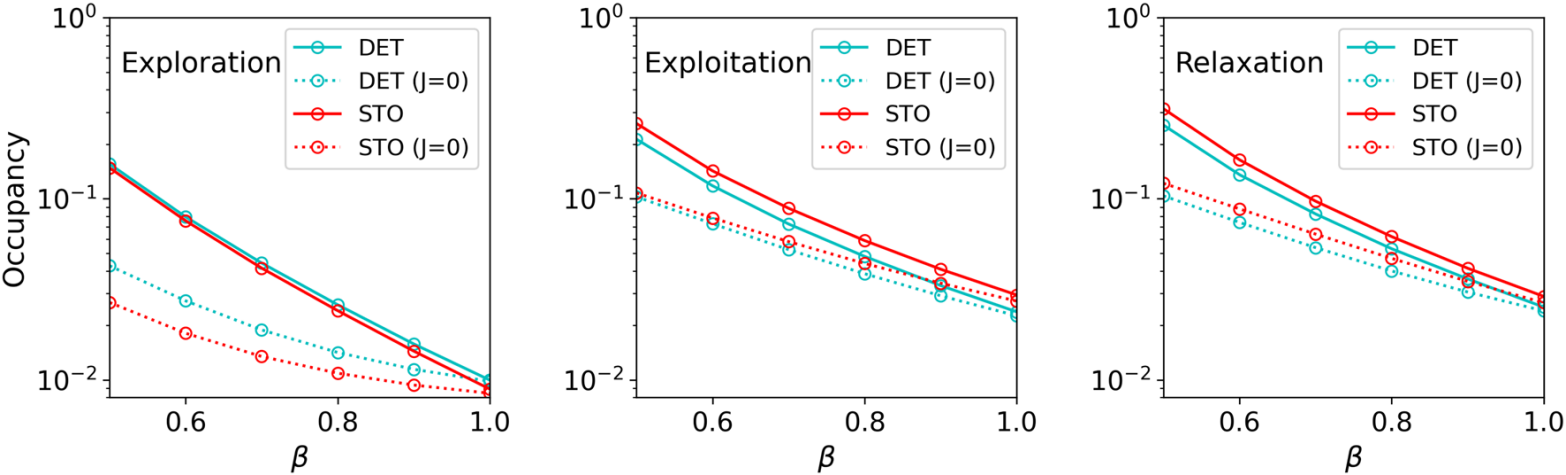
Overall occupancy (measured as the fraction of occupied nodes in the arena) as a function of the spin-glass parameter $\beta$ for the three phases of the experiment (exploration, exploitation, relaxation).

As depicted in Fig. 5, the overall occupancy of the *replicas* escalates as $\beta$ decreases across all experiment phases. Although differences in occupancy between general *replicas* and those with $J=0$ tend to widen with decreasing $\beta$, these differences are minimal as we approach our default case of $\beta =1$. Comparing the activities observed in the plots we can conclude that a fair comparison between general spin-glasses and the case $J=0$ can be obtained if using the default value $\beta =1$ for the former, and $\beta =0.9$ for the latter. Accordingly, all the results presented in the following Sections will be based on this choice; anyway, we stress that using $\beta =1$ in all cases would not significantly modify our conclusions. As a whole, this ensures that our results remain mostly unbiased by potential variations in overall activity/occupancy.

### Exploring the efficiency of ant collective foraging

The potential to generate computational replicas of colonies opens up avenues for conducting *in silico* experiments, allowing us to anticipate the behavior of real colonies in various hypothetical scenarios. Traditional experimental approaches can be often resource-intensive, expensive, or constrained by limited datasets. In contrast, computational models serve as valuable complements to experimental work, offering a cost-effective means to explore a broader range of conditions and providing guidance for designing and interpreting experiments. By employing *in silico* experiments, we can efficiently simulate and analyze colony behavior under diverse conditions, thereby enhancing our ability to predict and comprehend the complex dynamics of these systems.

Building on the insights gained from “Experimental patterns of ant foraging” , where the most significant differences between the spatial patterns under deterministic and stochastic food conditions were detected during the recruitment phase, we leverage a spin glass model tailored to this phase to scrutinize the impact of those correlation patterns on food detection and gathering efficiency. Specifically, we employ our spin-glass *replicas* of the colony, focusing on a simulated arena mirroring the structure of the real environment. To assess the effectiveness of these *replicas*, a crucial benchmark is established using a pertinent null model, given again by the *replicas* where spatial correlations are completely absent ($J=0$), to enable a thorough evaluation of how correlation patterns contribute to the efficiency.

The evaluation metric for foraging efficiency involves computing the time required for the *replicas* to identify the presence of the two food items located in the simulated arena, mimicking the real-world structure. This is quantified as the time at which the colony, in its *replica* form, has registered a transition from the empty state ($I_{i^{F}}=0$) to the occupied state ($I_{i^{F}}=1$) for all the nodes of the arena where the food is located (these are denoted by $i^{F}$). This particular measure of efficiency (which involves detection of all the food nodes present in the arena) has the virtue to combine the results of the initial exploration the arena together with a significant part of the gathering process, in agreement with our choice of the recruitment phase for training the *replicas*.

**Figure 6:**
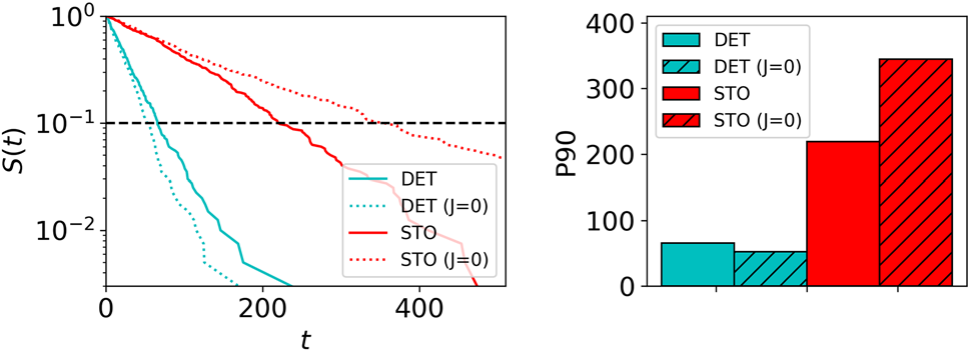
Foraging efficiency for the computational *replicas* of the colony (using the data from the exploitation phase for training) under the food conditions (deterministic/stochastic) to which each colony is trained. (Left): survival probability of the food up to time *t*. (Right): percentile 90 of the times to reach the first piece of food (the position of the percentile is also indicated in the left plot with a dashed horizontal line for the sake of clarity).

We﻿ conducted in silico simulations where the food locations $i^{F}$ corresponded to the actual food locations discovered by each colony-whether deterministic or stochastic-in our real-world experiments. This approach mirrors then our experimental conditions, but with the advantage of computationally executing hundreds or thousands of realizations, thereby enhancing statistical robustness. When simulating the *replicas* of the colonies under their respective food conditions, a noteworthy observation emerges: the deterministic colony consistently requires significantly less time to locate the food compared to its stochastic counterpart. This is evident in Fig. 6, where the left panel illustrate the survival probability of the process (indicating the likelihood that the food has not been reached yet after a certain time) and the right panel depicts the 90th percentile of the search time to reach the food. Notably, the impact of spatial correlations is nontrivial in this scenario. The replicas for $J = 0$ yield slightly better efficiencies for the deterministic colonies, but worse efficiencies for the stochastic case when compared to the case $J \ne 0$. This should be associated to the different behavior observed above between the two scenarios for the exploitation phase. The preconditioning induced by the deterministic scenario promotes a fast recruitment and food gathering focused on a relatively small region of the lattice, largely suppressing exploration and the capacity of the *replicas* to reach further regions. The stochastic scenario, instead, elicits a slower recruitment response and keeps exploration of new regions at higher levels. The fact that the stochastic scenario requires from the spatial correlations to keep search efficiency high, thus, tells us that collective organization of that exploration is essential, and so these correlations effectively introduce a nuanced layer of complexity. Instead, exploitation of the resources in the deterministic case does not require from such correlations, as that behavior is much more systematic and can be mostly explained by independent occupancy of the different regions connecting the nest to the food. Actually, the fact that efficiency even increases for the case $J=0$ probably means that some (small) negative spatial correlations are present in the system (maybe because of some crossing effects between the paths to the two food patches).

### Impact of resource spatial unpredictability

In the subsequent analysis, we consider a theoretical scenario in which food locations $i^{F}$ adhere to the deterministic experimental condition with a probability of *p*, while with a complementary probability of $1-p$, these locations are selected randomly. This setup enables a comparison of the performance of *replicas* generated from deterministic versus stochastic datasets. The objective is to assess how these *replicas* respond to food conditions differing from those to which they are initially trained.

**Figure 7:**
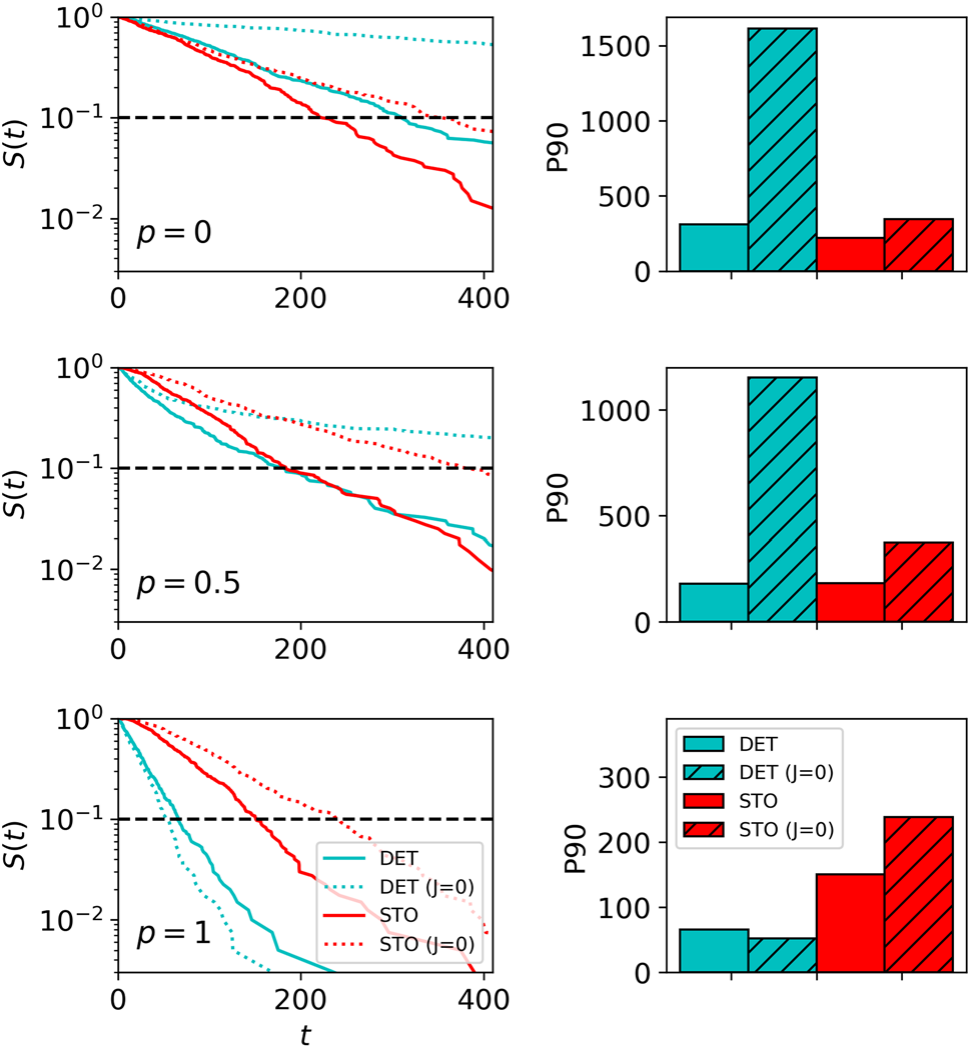
Foraging efficiency, measured as the time spent finding the resource,for the computational *replicas* of the colony in the different (deterministic/stochastic) food conditions. The training was done with the dataset from the exploitation phase. The first column shows the survival probability of the food up to time *t* for the replicas simulating the recruitment phase. We compare the cases of $p=0$, 0.5, 1 (first, second and third row, respectively), where *p* is the probability of locating the food patches at the same place as in the deterministic scenario.The second column shows the corresponding search efficiency measured as the percentile 90 of the times to reach the food (the position of the percentile is also indicated in the left plots with a dashed horizontal line for the sake of clarity).

The results, again using the exploitation phase dataset for training the spin-glass, are depicted in Fig. 7. Notably, there is a stark decline in the efficiency observed for the deterministic colony when *p* decreases, and so it is faced with unfamiliar food conditions (as shown in the third row of Fig. 7). So, in the intermediate case ($p=0.5$) both *replicas* reach a very similar efficiency (second row). Instead, the times required to detect food for the stochastic *replica* when *p* is small (first row) do not decrease significantly provided that correlations are considered, so this *replica* readily adapts to the deterministic condition. This substantiates our confirmation that a colony trained and parameterized to exploit deterministic resources exhibits diminished resilience or adaptability to external changes compared to the stochastic preconditioning, particularly in terms of food location. Note that the stochastic case without correlations ($J=0$) does not exhibit such resilience, so revealing that correlations are an important part of the collective adaptation mechanism used by the colony to sustain its foraging efficiency under flexible conditions.

It is significant that, in contrast to the outcomes in Fig. 6, the results under uncertain/random food conditions (this is, for *p* small) now display a consistent trend across both food conditions. Specifically, the survival distribution for the *replica* without correlations (case $J=0$) decays significantly more slowly than for the more general case (i.e. $J>0$) for both the deterministic and stochastic scenarios. The presence of correlations, once again, proves advantageous as it promotes the simultaneous occupancy of diverse regions within the arena. This organizational advantage becomes particularly beneficial in scenarios where food is situated in distant or new/challenging places for the colony to be reached. The observed trends provide then direct evidence of the pivotal role spatial correlations play in enhancing foraging efficiency under conditions of uncertainty.

### Impact of the resource distance to the nest

While we have asserted that the increased efficiency of *replicas* under stochastic conditions is attributed to correlations and/or collective effects, one could argue that, in reality, the *replica* trained under stochastic conditions might simply be more inclined to reach further regions of the arena. This argument suggests that, by chance, some experiments placed food in those distant nodes, thereby explaining the lower efficiency of *deterministic replicas* for small *p* values and higher efficiency for large *p* values.

**Figure 8:**
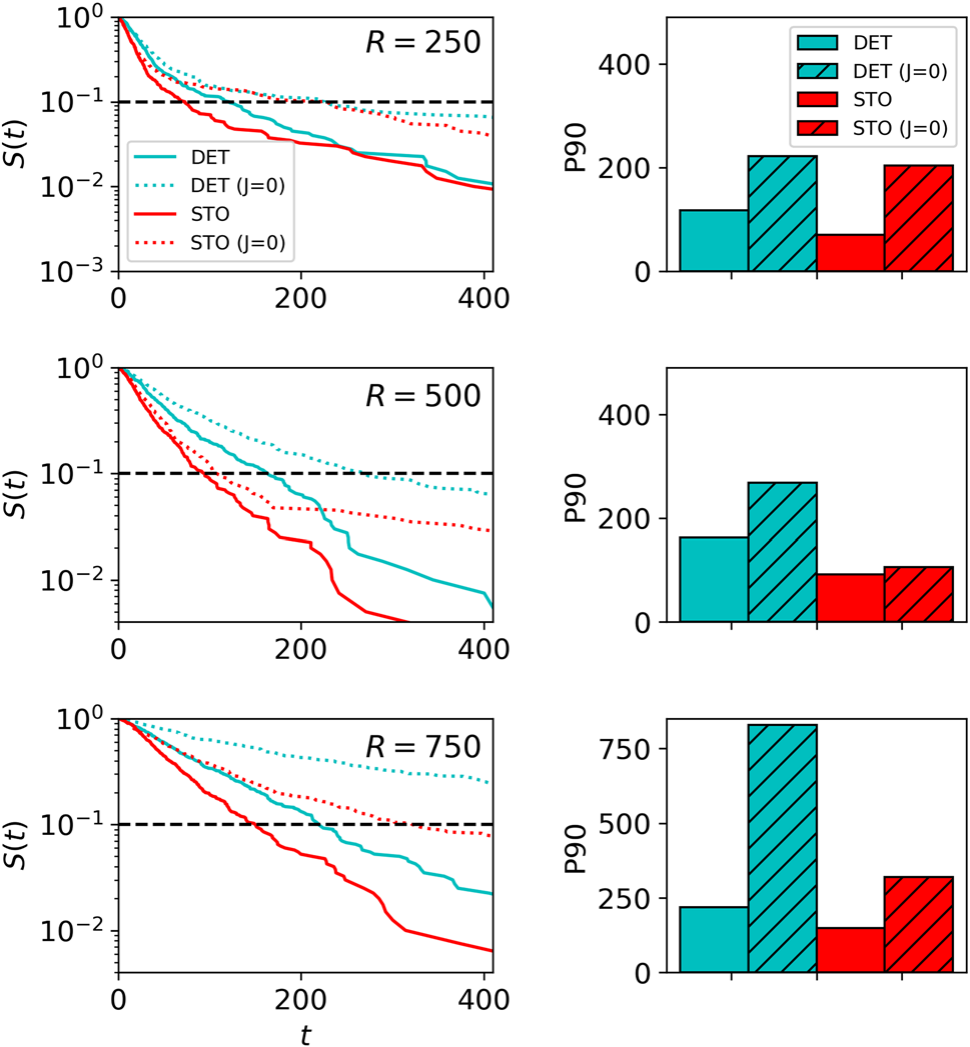
Foraging efficiency, measured as time spent finding the resource, for the computational replicas of the colony as a function of the distance between the nest and the food. In the first column, we compare the survival probability of the food up to time *t* for the replicas in the phases of Recruitment, for the cases of $R=250$, 550, 750 mm (first, second and third row, respectively). The second column shows the corresponding search efficiency measured as the percentile 90 of the times to reach the food (the position of the percentile is also indicated in the left plot with a dashed horizontal line for the sake of clarity).

To eliminate this alternative interpretation, we consider a scenario where the food location $i^{F}$ is randomly generated, but only within a radial distance *R* from the nest, with varying values of *R*. Considering that deterministic food locations were approximately $\approx 500$ mm away from the nest, one might expect that for *R* values below this threshold, the efficiency of both replicas (stochastic and deterministic) should be equivalent. However, our results in Fig. 8 refute this hypothesis, confirming that, regardless of the value of *R*, stochastic replicas consistently demonstrate higher efficiency in detecting randomly located food (particularly evident in the $R=500$ case; Fig. 8 middle row panels). Additionally, Fig. 8 reiterates that colony replicas in the general case (i.e. $J>0$) are significantly more efficient than those for the $J=0$ case. Consequently, the notion that stochastic colony replicas consistently exhibit better adaptability to uncertain scenarios, due to spatial pairwise correlations, appears to be validated.

## Discussion

The issue of pattern and scale, often posited as a central challenge in ecology ^48^, serves to bridge the realms of behavioural ecology and population biology. This concern is equally important in the realm of sociality, particularly in examining the interplay between individual behavior and the formation of collectives or groups. In this overarching framework, concepts derived from complexity science and information theory serve as invaluable tools for pinpointing relevant scales and underlying mechanisms.

Our work emphasizes on the adaptive and fluid nature of ant interactions and networks, underscoring the highly non-stationary character of collective foraging when properly integrating the natural spatiotemporal scales of the process. This stands in contrast to many theoretical models and theories related to collective motion, which often assume stationary conditions for the sake of simplicity. Furthermore, we quantify how conditioning collectives to specific foraging environments governs both the efficiency and flexibility of colonies.

From a more technical standpoint, the study demonstrates (i) that mutual information (i.e. pairwise correlations) can identify and quantify essential foraging patterns, mainly in the food gathering (exploitation phase), whereas in other contexts higher-order correlations might be also relevant, (ii) how a spin glass model, which essentially assumes pairwise correlations, can reproduce the resulting dynamics through computational replicas of ant colonies gathering food. Furthermore, our method allows to control for biases in colony behaviour derived from the fact that the number of ants changes with time. We do so by tuning a single parameter ($\beta$), revealing the spin glass framework as a flexible and easy-to-handle tool for conducting in-silico experiments. Indeed, spin glasses can serve as powerful predictive tools, overcoming various limitations associated with real experiments. In particular, they encapsulate key occupancy and spatial clustering patterns that impact on colony robustness and efficiency when collecting food in uncertain environments. Because of that, spin glasses can bring comprehensive understanding on how information transfer and movement patterns impact the adaptability and efficiency of foraging processes at the colony level.

As a proof of concept, we have delved into how the occupancy and correlation patterns of food-gathering ant colonies adjust to patterns of resource distribution. Our analysis suggests that uncertainty in resource distribution is likely to substantially alter the relative efficiency of various pre-conditioned foraging strategies. More specifically, colony *replicas* trained and parameterized to gather food in stochastic environments are more robust and effective to gather food in very different conditions compared to colony replicates particularly suited/adapted to one single food gathering condition. Remarkably, the difference observed is driven by spatial pairwise correlations, rather than the number of ants out in the arena.

In summary, we have exemplified how concepts and tools from information theory and statistical physics can be employed to quantitatively explore questions pertaining to the emergent and complex behavior of social dynamics. To do so, the analysis must properly consider all the temporal and spatial scales involved, accommodate concepts such as non-stationarity and the superposition of foraging modes within the analysis to understand and assess foraging complexity as a whole.

### Supplementary Information

Supplementary Information 1.

Supplementary Information 2.

Supplementary Information 3.

Supplementary Information 4.

Supplementary Information 5.

## Data Availability

All experimental data necessary to reproduce the results of the work is provided as part of the Supplementary Information files.
