# Supplementary material for: Spatiotemporal organization of ant foraging from a complex systems perspective
Source: Sci Rep. 2024 Jun 4;14:12801. doi: 10.1038/s41598-024-63307-1 (PMC11150503; doi:10.1038/s41598-024-63307-1)
Supplement: Supplementary file 1 — Supplementary Information 1. [file 41598_2024_63307_MOESM1_ESM.pdf]

Spatiotemporal Organization of Ant Foraging from a Complex Systems Perspective SUPPLEMENTARY MATERIAL

Javier Cristín[1,2,3], Pol Fernández-López[4], Roger Lloret-Cabot[4], Meritxell Genovart[4], Viçenc Méndez[3], Frederic Bartumeus[4,5], and Daniel Campos[3*]

[1] *Istituto Sistemi Complessi, Consiglio Nazionale delle Ricerche, UOS Sapienza, 00185 Rome, Italy*

[2] *Dipartimento di Fisica, Universita' Sapienza, 00185 Rome, Italy*

[3] *Grup de Física Estadística, Departament de Física. Facultat de Ciències), Universitat Autònoma de Barcelona, 08193 Bellaterra (Barcelona), Spain*

[4] *Centre d'Estudis Avançats de Blanes (CEAB-CSIC), Blanes Girona, Spain; CREAF, Cerdanyola del Vallès, Spain*

[5] *ICREA, Institut Català de Recerca i Estudis Avançats, Barcelona, Spain.*

(Dated: May 15, 2024)

S1. EXPERIMENTAL DATA PROCESSING: ACTIVITY AND INTERACTIONS

The occupancy of the nodes in the lattice was computed from the analysis of videos through standard imaging libraries in Python language. We define a radius R_a. When an ant is detected at a lower distance than R_a from the center of a certain i-node in a given frame, we assign the ant to that i-node in that given frame. We have checked the value of R_a to avoid overlaps between nodes and sufficiently high to capture the spatial patterns. The optimal value of R_a is related with the hexagonal structure and the characteristic distance between the nodes. We have used a value $R_a = 20mm$ (we have checked small modifications over the range 10 to $40mm$ does not affect qualitatively the results).

Once the number of ants detected in each node of the lattice was determined for each frame of the video. If at least one ant is detected at the i-node in a certain frame, we assign a value $I_i = 1$ (occupied) to the node at that time. Otherwise, the value is set to 0 (empty). So we transform the videos to a binary-state signal of occupation for each node, with a framerate of 2Hz. Also, if there is more than one ant at the i-node in a certain frame, then an interaction occurs at that node.

A. Relative number of interactions in the experiments

The results shown in the plots below (Figures S1 and S2) for the number of interactions and the occupancy of the arena "per capita" (this is, divided by the number of ants in the arena) complement the information in the Section IIIA in the main manuscript. As can be seen, ants tend to interact more in DET experiments during the exploitation and relaxation phases, as opposed to the exploration phase. We also observed that the occupancy "per capita" (i.e proportion of nodes occupied divided by the number of ants) is larger during the exploration phase in the DET experiment compared to the STO ones. However, no differences seemed to occur in the occupancy "per capita" for the exploitation and relaxation phases, despite the variability being much larger in the DET scenarios.

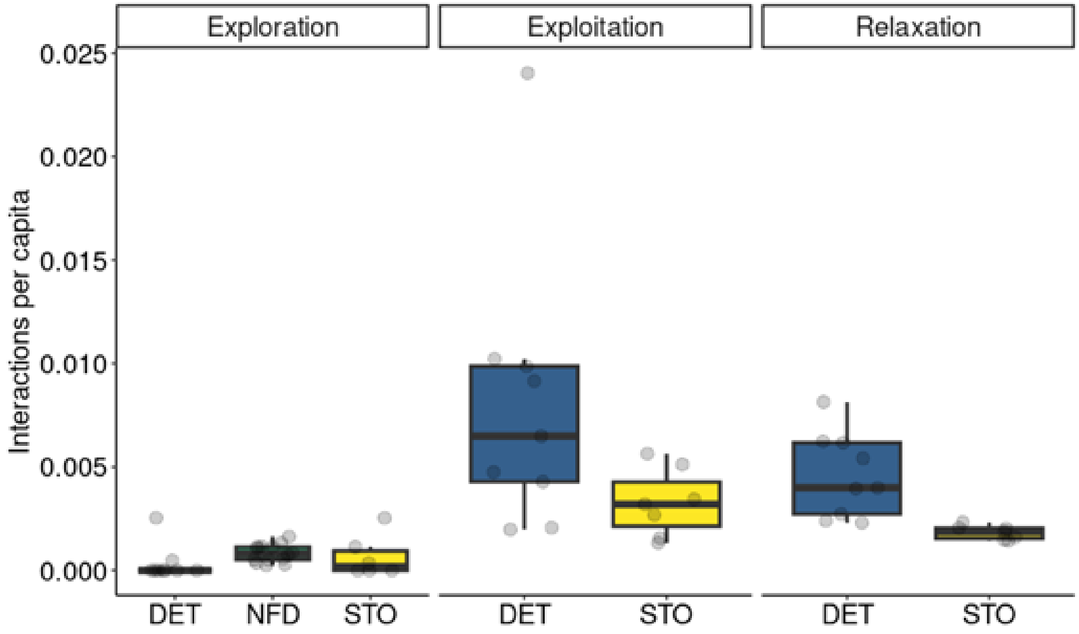

FIG. S1. Average interactions "per capita" measured as the proportion of the number of ant-ant crossings divided by the number of ants in the arena at each frame, and then averaging for each phase and condition. Note that the NFD (No Food) condition only involves exploration, and therefore only appears in the first panel.

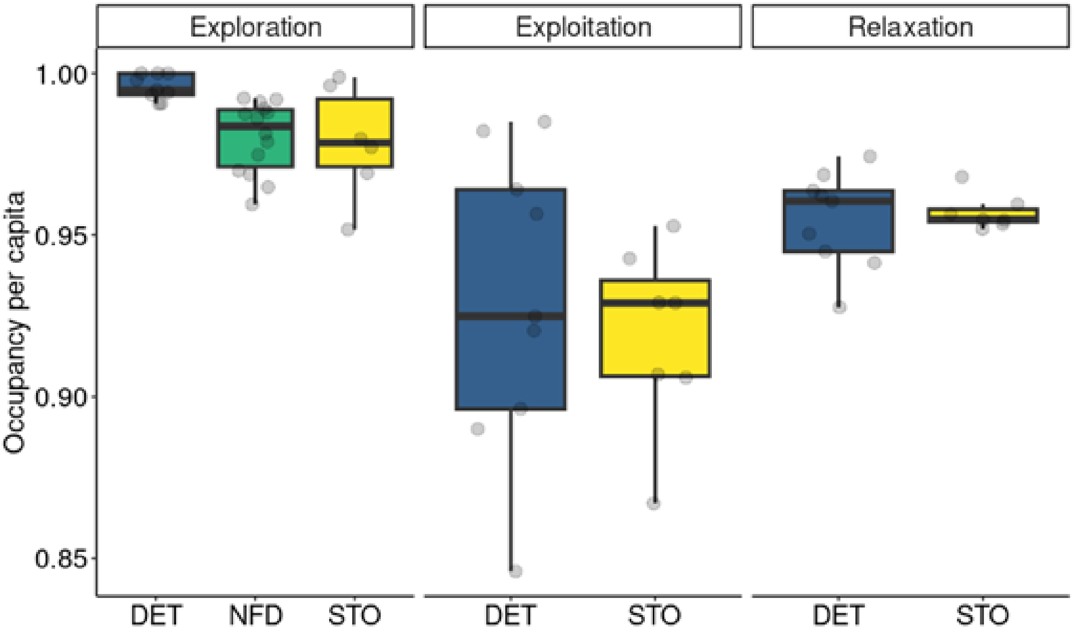

FIG. S2. Average occupancy "per capita" measured as the proportion of the number of nodes occupied divided by the number of ants in the arena at each frame, and then averaging for each phase and condition. Again, the NFD (No Food) condition only involves exploration, and therefore only appears in the first panel.

S2. PARAMETERIZATION OF THE INVERSE SPIN-GLASSES IN DIFFERENT REGIONS/PHASES

The parameters h_i, J_{ij} present in the Hamiltonian of the spin-glass (see Eq. (6) of the main manuscript) are inferred through pseudo-likelihood methods (see [1, 2]). Note that the model contains N parameters of type h_i and $N(N-1)$ parameters of type J_{ij} , for a total of N^2 parameters in the system (with $N = 620$ if we consider all nodes in the experimental arena).

The parameters h_i carry information about the mean occupancy of the i-th node/region, while J_{ij} is related to the couplings (or correlations) between the occupancies of the nodes i and j. So that, their inferred values in general give us information about the spatial distribution of foraging and its spatial organization (see [3] for a complete discussion). Furthermore, applying the spin-glass model inference to just some (spatial or temporal) specific parts of the occupancy dataset, we can obtain particular information about those specific part and/or compare them.

In Table S1, for instance, we compare the effect of considering the whole datasets obtained from the experiments (whose average occupancy is denoted by $\langle h \rangle$) to those where we only consider the region close to (i) food/targets and (ii) to the nest (with $\langle h_f \rangle$ and $\langle h_n \rangle$ being the averages of h_i computed on that regions, respectively). The same applies to the averages over the coupling parameters (denoted by $\langle J \rangle$, $\langle J_f \rangle$ and $\langle J_n \rangle$, respectively).

Note that in this case a direct comparison between values $\langle h \rangle$, $\langle h_f \rangle$ and $\langle h_n \rangle$ is not sound since the size of the corresponding regions is different, and this can modify the parameter values in a non-trivial way. Instead, we use these values to check how they change between the different food conditions (deterministic or stochastic) under which they have been foraging, and how they would adapt when computational simulations on the other food scenario (deterministic or stochastic) are conducted. So, in Table S1 the notation *Det-fSto* refers to the case where we take the dataset of the colony that has been foraging under deterministic conditions, but we compute $\langle h_f \rangle$ as the mean occupancy in the region where the food was placed for the other (stochastic) scenario.

The corresponding results show that both the average occupancies and coupling parameters near the food ($\langle h_f \rangle$ and $\langle J_f \rangle$, respectively) increase when the scenario used corresponds to the real conditions under which the colonies have been foraging. This is an additional indirect checking that the *replicas* generated through the spin-glass approach show a response which is adapted to the real conditions of the corresponding real colony.

	General Parameters					
	$\langle h\rangle$	$\langle h_f\rangle$	$\langle h_n\rangle$	$\langle J\rangle$	$\langle J_f\rangle$	$\langle J_n\rangle$
Det-fDet	-0.050 ± 0.001	-0.035 ± 0.008	-0.027 ± 0.005	0.0017 ± 0.00003	0.0009 ± 0.0002	0.0019 ± 0.0002
Det-fSto	-0.050 ± 0.001	-0.0516 ± 0.0006	-0.027 ± 0.005	0.0017 ± 0.00003	0.0018 ± 0.00005	0.0019 ± 0.0002
Sto-fDet	-0.048 ± 0.0003	-0.0490 ± 0.0005	-0.021 ± 0.006	0.0016 ± 0.00002	0.0016 ± 0.00001	0.0050 ± 0.0004
Sto-fSto	-0.048 ± 0.0003	-0.0476 ± 0.0008	-0.021 ± 0.006	0.0016 ± 0.00002	0.0014 ± 0.0005	0.0049 ± 0.0004

TABLE S1. *Averaged external field and pairwise interaction for the patches that contain resources ($\langle h_f\rangle$ and $\langle J_f\rangle$), for the entire structure ($\langle h\rangle$ and $\langle J\rangle$) and for the region close to the nest ($\langle h_n\rangle$ and $\langle J_n\rangle$). The results are presented for the colony A and both experimental conditions in the exploitation phase.*

	Analyzing the temporal evolution of h					
	Exploitation			Relaxation		
	$\langle h\rangle$	$\langle h_f\rangle$	$\langle h_n\rangle$	$\langle h\rangle$	$\langle h_f\rangle$	$\langle h_n\rangle$
Det-fDet	-0.050 ± 0.001	-0.035 ± 0.008	-0.027 ± 0.005	-0.048 ± 0.0002	-0.034 ± 0.001	-0.024 ± 0.005
Det-fSto	-0.050 ± 0.001	-0.0516 ± 0.0006	-0.027 ± 0.005	-0.048 ± 0.0002	-0.049 ± 0.0006	-0.024 ± 0.005
Sto-fDet	-0.048 ± 0.0003	-0.0490 ± 0.0005	-0.021 ± 0.006	-0.0464 ± 0.0002	-0.0487 ± 0.0005	-0.023 ± 0.006
Sto-fSto	-0.048 ± 0.0003	-0.0476 ± 0.0008	-0.021 ± 0.006	-0.0464 ± 0.0002	-0.0462 ± 0.0004	-0.023 ± 0.006

TABLE S2. *Averaged external field for the patches that contain resources ($\langle h_f\rangle$), the averaged field for the entire structure ($\langle h\rangle$) and the averaged field close to the nest ($\langle h_n\rangle$). The results are presented for the colony A and both experimental conditions. The values are divided into two regimes, each one of them coming from a part of the temporal signal of the experiments.*

S3. INVERSE SPIN-GLASS MODELS PROVIDE RELIABLE *REPLICAS* OF THE COLONY DYNAMICS

If we apply the spin-glass models to generate *replicas* reproducing the patterns in the different phases of the foraging process (*exploration*, *exploitation*, *relaxation*), we can use them to investigate the temporal evolution of the system. For this purpose, we have compared the main properties of the set of h_i and J_{ij} parameters obtained. However, since occupancies in the *exploration* phase are obviously low (specially in some particular realizations of the experiments) we only show the results for the *exploitation* and the *relaxation* phases; the results are presented in Tables S2 and S4. We observe that the mean values of the spin-glass parameters (both $\langle h\rangle$ and $\langle J\rangle$), and their corresponding dispersion, show a clear tendency in all cases. The mean occupancy $\langle h\rangle$, in particular, is consistently larger in the *relaxation* period, since on average the occupancy during this phase is also higher, and the same is found for the occupancies around the food. For the nodes around the nest, however, the colony is specially active in this region during the *exploitation* phase and so this is reflected in the values of $\langle h_n\rangle$. Regarding the intensity of correlations (computed through $\langle J\rangle$), on the contrary, they decrease during the *relaxation* phase if compared to the *exploration+exploitation* one. This is because such correlations (as happens with the mutual information computed in Fig. 4 of the Main Text) are specially reflecting the presence of the paths to food and similar collective patterns, so these will be apparent during the *exploitation* phase. These collective effects seems to be stronger around the nest, while they become almost negligible in the region around the food (probably because a significant part of this region is not part of the nest-food paths set by the ants).

Note that replicas obtained from the spin-glass approach cannot reproduce the rich detail of the dynamics in the evolution of the occupancy as a function of time (e.g. as done in Figure 2 of the main manuscript). This is because such approaches only capture stationary average behavior of the colony. So that, if we generate spatial occupancy patterns using the replicas (starting from an initial condition where all nodes are empty, mimicking the situation used in our experimental setup) then we will observe that the occupancy increases monotonically until reaching a saturation state where it simply fluctuates around this stationary behavior. The situation is illustrated in Fig. S3.

To quantify how well the occupancy states from the computational *replicas* capture the correlations present in the experimental system we have followed the methodology in [4], which consists of building the distribution of occupancy states $\boldsymbol{I}$ in the experimental system; this is denoted by $P_{exp}^{1:N}(\boldsymbol{I})$. The corresponding Shannon entropy for this distribution reads then $S_{exp} \equiv -P_{exp}^{1:N}(\boldsymbol{I})\left[\log P_{exp}^{1:N}(\boldsymbol{I})\right]$. Similarly, one can compute the equivalent entropy obtained from the synthetic trajectories (we denote these by S_s and S_{s0} for the general case and the case $J = 0$, respectively). Then, since spin-glasses can only implement pairwise correlations, comparing the entropy obtained from the general spin-glass model to the experimental value we obtain the amount of information $H_1 = S_s - S_{exp}$ present in the system that cannot be captured by pairwise correlations and, similarly, $H_0 = S_{s0} - S_{exp}$ represent the amount of information lost when correlations are completely neglected in the system. So that, the quotient H_1/H_0 provides a proper measure of the fraction of the information that can be captured through considering pairwise (but

	Analyzing the temporal evolution of J					
	Exploitation			Relaxation		
	$\langle J \rangle$	$\langle J_f \rangle$	$\langle J_n \rangle$	$\langle J \rangle$	$\langle J_f \rangle$	$\langle J_n \rangle$
Det-hDet	0.0017 ± 0.00003	0.0009 ± 0.0002	0.0019 ± 0.0002	0.0017 ± 0.0001	0.0009 ± 0.0001	0.0011 ± 0.00008
Det-hSto	0.0017 ± 0.00003	0.0018 ± 0.00005	0.0019 ± 0.0002	0.0017 ± 0.0001	0.0018 ± 0.0002	0.0011 ± 0.00005
Sto-hDet	0.0016 ± 0.00002	0.0016 ± 0.0001	0.0050 ± 0.0004	0.0016 ± 0.00003	0.0016 ± 0.0001	0.0008 ± 0.00006
Sto-hSto	0.0016 ± 0.00002	0.0014 ± 0.0005	0.0049 ± 0.0004	0.0016 ± 0.00003	0.0015 ± 0.00005	0.0008 ± 0.00006

TABLE S3. *Averaged pairwise interaction for the patches that contain resources ($\langle J_f \rangle$), the averaged interaction for the entire structure ($\langle J \rangle$) and the averaged interaction close to the nest ($\langle J_n \rangle$). To do the average, only the interaction with patches located at a smaller distance of* 200*mm are taken. The results are presented for the colony A and both experimental conditions. The values are divided into two regimes, each one of them coming from a part of the temporal signal of the experiments.*

	Analyzing the temporal evolution of J					
	Exploration + exploitation			Relaxation		
	$\langle J \rangle$	$\langle J_f \rangle$	$\langle J_n \rangle$	$\langle J \rangle$	$\langle J_f \rangle$	$\langle J_n \rangle$
Det-hDet	0.70 ± 0.33	-0.07 ± 0.06	0.01 ± 0.04	1.66 ± 0.57	0.03 ± 0.06	0.02 ± 0.05
Det-hSto	0.22 ± 0.32	0.06 ± 0.21	0.01 ± 0.04	0.23 ± 0.43	0.11 ± 0.20	0.01 ± 0.03
Sto-hDet	2.30 ± 0.80	-0.15 ± 0.06	0.08 ± 0.07	3.81 ± 1.30	0.14 ± 0.12	0.07 ± 0.07
Sto-hSto	0.27 ± 0.12	0.01 ± 0.09	0.04 ± 0.04	0.43 ± 0.28	-0.01 ± 0.06	0.05 ± 0.04

TABLE S4. *Averaged pairwise interaction for the patches that contain resources ($\langle J_f \rangle$), the averaged interaction for the entire structure ($\langle J \rangle$) and the averaged interaction close to the nest ($\langle J_n \rangle$). To do the average, only the interaction with patches located at a smaller distance of* 200*mm are taken. The results are presented for both colonies and both experimental conditions. The values are divided into two regimes, each one of them coming from a part of the temporal signal of the experiments.*

not higher-order) correlations.

In our case, when we compute the measures above using the experimental dataset and our *replicas* we find that H_1/H_0 takes a value of 0.65 when considering the whole experimental datasets, while using only the data from particular phases or food conditions the values go from 0.40 (obtained for the exploration phase, where higher fluctuations between the different experimental realizations appear) to 0.96 (for the relaxation phase, where a more homogeneous occupancy of the arena is observed, and then the entropies above in general show smaller values. For the *exploitation* case, correspondingly, an intermediate value of 0.65 is found. So, we can assert that pairwise correlations represent a significant amount of the correlations observed in the real system, while this becomes particularly reliable for the latest parts of the experiments.

For the sake of completeness, we also show how pairwise correlations obtained from the experiments compare to those from the synthetic trajectories. Figure S4, in particular, summarizes the results found when comparing the average mutual information between (i) experimental trajectories, (ii) synthetic trajectories from the general spin-glasses, and (iii) synthetic trajectories for the spin-glass where correlations are absent, $J = 0$. For all food conditions (deterministic, stochastic, and the control case without food) the synthetic trajectories for the general case capture with a high level of accuracy the statistical patterns of pairwise correlations exhibited by the experimental trajectories, so confirming again the goodness of the spin-glasses as *replicas* of the real colonies. Instead, the case $J = 0$ is unable to reproduce such dynamics (actually, by definition the case $J = 0$ would ideally yield $\langle \mathrm{MI} \rangle = T(\boldsymbol{I}) = 0$, but the noise involved in the process to generate the synthetic trajectories make that at practice some small correlations do still persist).

Alternatively, the temporal dynamics predicted from our computational (spin-glass) *replicas* can be compared to the patterns of the real colony by measuring the distribution of persistence times τ (defined as the amount of time one node stays in the same state, either occupied $I_i = 1$ or empty $I_i = 0$, before switching to the other one), as seen in [3]. These times provide information about the dynamics of fluctuations in the occupancy patterns. Experimental distributions of τ are found to follow a non-trivial behavior which corresponds to an intermediate decay between exponential and power-law functions (figure S5). Although it is not possible to derive a simple analytical expression for that distribution, we observe that the *replicas* show again a very good agreement with the experimental data. So, these results confirm that the spin-glass method is able to reproduce dynamical properties of the colony, too, as well as the average properties of the experimental occupancy patterns.

[1] A. Hyvarinen. *Consistency of pseudolikelihood estimation of fully visible Boltzmann machines.* Neural Comp, 18, 2283 (2006).

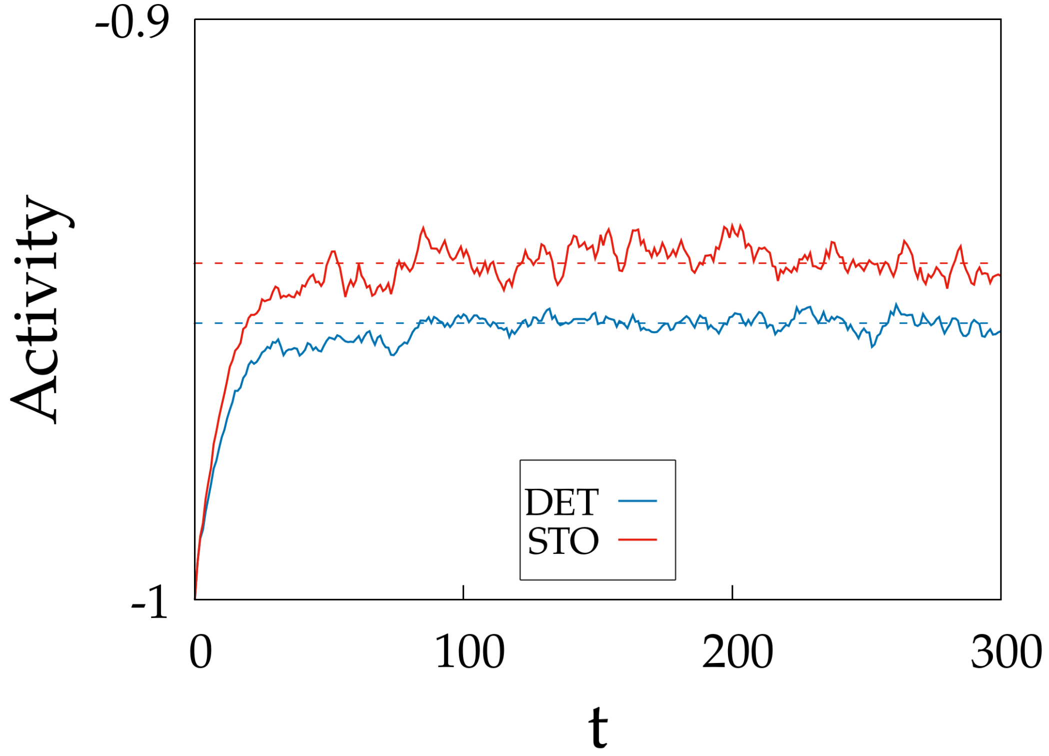

FIG. S3. Time evolution of the occupancy generated computationally through the SG approach in the same case as used in Sections (this is, using the exploitation phase as a dataset for training the replicas). The saturation observed after a transient time corresponds to the stationary situation that the SGs generate as an average pattern mimicking the properties of the training dataset.

[2] N.C. Nguyen, R. Zecchina and J. Berg. *Inverse statistical problems: from the inverse Ising problem to data science*. Adv. Phys. 66, 197 (2017).

[3] Cristín J., Bartumeus F., Méndez V. and Campos, D. *Occupancy patterns in superorganisms: a spin-glass approach to ant exploration*. R. Soc. Open Sci. 7:201250 (2020).

[4] C.W. Lynn, L. Papadopoulos, D.D. Lee and D.S. Bassett. *Surges of Collective Human Activity Emerge from Simple Pairwise Correlations*. Phys. Rev. X 9, 011022 (2019).

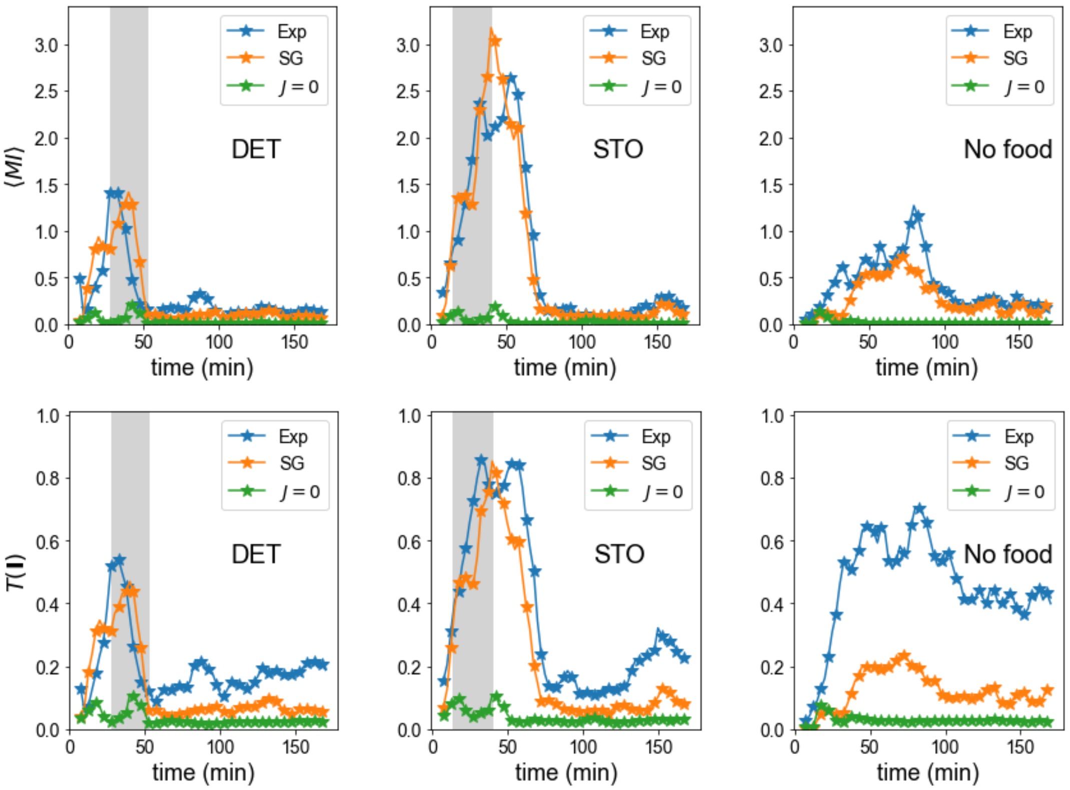

FIG. S4. Comparison of the time evolution of the average mutual information (upper panels) and the total correlations (lower panels) for experimental trajectories (Exp.) against synthetic trajectories generated through the spin-glass framework in the general case (SG) and in the case with correlations suppressed ($J = 0$). The results are presented (see legends) for the different food conditions (deterministic, stochastic, or the control case without food). Solid lines between points are included just to facilitate visualization, and the recruitment phase is marked (in shaded grey) to facilitate the interpretation. In all cases, the results are obtained as an average over a moving time window of 10 minutes centered at each specific time, and then averaging over the different experiments.

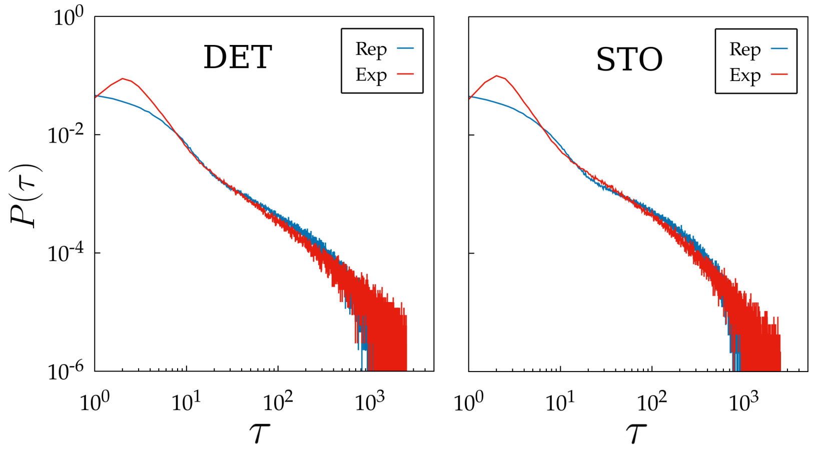

FIG. S5. Probability distribution of the persistence time $P(\tau)$) for the colony A in the a) Deterministic conditions and b) Stochastic conditions, computed from the experimental data ('Exp', red line) and from the simulated replicas ('Rep', blue line).
